# TNAP: A New Multitask Enzyme in Energy Metabolism

**DOI:** 10.3390/ijms221910470

**Published:** 2021-09-28

**Authors:** Anne Briolay, Laurence Bessueille, David Magne

**Affiliations:** University of Lyon, UCBL, CNRS, UMR 5246, ICBMS, 69622 Villeurbanne, France; Anne.briolay@univ-lyon1.fr (A.B.); laurence.bessueille@univ-lyon1.fr (L.B.)

**Keywords:** TNAP, metabolic syndrome, liver, bile, steatosis, adipocyte, lipopolysaccharide, CD36, phosphocreatine

## Abstract

Tissue-nonspecific alkaline phosphatase (TNAP) is mainly known for its necessary role in skeletal and dental mineralization, which relies on the hydrolysis of the mineralization inhibitor inorganic pyrophosphate (PP_i_). Mutations in the gene encoding TNAP leading to severe hypophosphatasia result in strongly reduced mineralization and perinatal death. Fortunately, the relatively recent development of a recombinant TNAP with a bone anchor has allowed to correct the bone defects and prolong the life of affected babies and children. Researches on TNAP must however not be slowed down, because accumulating evidence indicates that TNAP activation in individuals with metabolic syndrome (MetS) is associated with enhanced cardiovascular mortality, presumably in relation with cardiovascular calcification. On the other hand, TNAP appears to be necessary to prevent the development of steatohepatitis in mice, suggesting that TNAP plays protective roles. The aim of the present review is to highlight the known or suspected functions of TNAP in energy metabolism that may be associated with the development of MetS. The location of TNAP in liver and its function in bile excretion, lipopolysaccharide (LPS) detoxification and fatty acid transport will be presented. The expression and function of TNAP in adipocyte differentiation and thermogenesis will also be discussed. Given that TNAP is a tissue- and substrate-nonspecific phosphatase, we believe that it exerts several crucial pathophysiological functions that are just beginning to be discovered.

## 1. Introduction

Tissue-nonspecific alkaline phosphatase (TNAP) is together with intestinal AP (IAP) the only AP that is expressed in humans after birth (reviewed in [1]). With few exceptions detailed below, it is moreover the only AP that has its active site inside the body, since IAP is active in the intestinal lumen and only occasionally found in the blood (discussed below). As its name indicates, TNAP is expressed in multiple tissues such as bone, growth plate cartilage, tooth, liver, kidney or brain [1]. In most cell types, it is attached to the cell membrane by a glycosylphosphatidylinositol (GPI) anchor. In some cells and in different circumstances, TNAP can be released extracellularly, either attached to the membrane of extracellular vesicles, or as a soluble enzyme after the cleavage of the GPI anchor. While circulating TNAP mainly originates from bones during skeletal growth, it comes from both bones and the liver in adults [2]. In adulthood, strong increases in serum TNAP activity often reflect cholestasis, either intrahepatic or extrahepatic, or biliary obstruction due to cancer [3]. More modest elevations occur in the context of hepatitis, and more generally in individuals with metabolic syndrome (MetS), in whom serum TNAP levels predict cardiovascular mortality [4,5,6,7,8,9]. Since the main function of TNAP in humans is to drive tissue mineralization [by dephosphorylating the mineralization inhibitor inorganic pyrophosphate (PP_i_)], it is suspected that the association between TNAP levels and cardiovascular mortality relies on the exacerbation of atherosclerosis plaque calcification [10]. The hypothesis that TNAP activation in patients with MetS worsens the cardiovascular risk by increasing vascular calcification has been recently reviewed [11] and is not the subject of the present article. Instead, we review here published data providing evidence to better understand why TNAP levels are increased in adults with MetS. Since the liver is the main source of circulating TNAP in MetS, and TNAP is expressed by hepatocytes and cholangiocytes [12,13], we will first explore the pathophysiological function of TNAP in the liver, the bile and the intestinal lumen. Then, we will move to recent articles suggesting unanticipated functions of TNAP in adipocytes.

## 2. TNAP’s Pathophysiological Functions in the Liver, Bile and Intestinal Lumen

The group of Olga Martinez-Augustin, in this journal issue, reported that *Alpl*^+/−^ mice develop liver steatosis when fed a high fat diet (HFD) [14]. This suggests that at least in mice, TNAP exerts metabolic functions. Whether this is also true in humans is unknown and difficult to investigate. Individuals with TNAP deficiency (hypophosphatasia, HPP) have not been reported to develop metabolic diseases [15,16]. This does not necessarily mean that TNAP deficiency has metabolic consequences in mice but not in humans. HPP is a rare disease, which makes virtually impossible to design clinical studies with a sufficient number of HPP patients. Moreover, children with severe HPP, who, a decade ago died in early life from bone hypomineralization, are now increasingly treated with recombinant asfotase alfa [17]. This recombinant TNAP is designed to attach to bone, but it is present in high levels in the circulation in treated children, where it likely exerts most of the extraosseous functions of TNAP and prevents the development of metabolic diseases. In this chapter, we will nevertheless present several studies performed with cultured human cells, which suggest that in humans like in mice, TNAP has metabolic functions.

### 2.1. Localization of TNAP in the Liver

**Figure 1:**
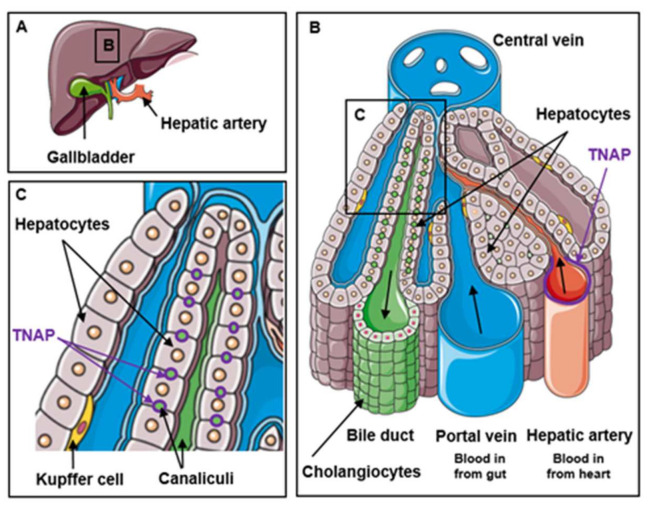
Location of TNAP in the liver. (**A**) Rough presentation of the liver structure indicating the region presented in (**B**). (**B**) Typical organization of hepatocytes and cholangiocytes delimitating bile ducts and liver vascularization showing TNAP location in hepatic artery. (**C**) Detail of (**B**) showing TNAP location in hepatocyte membranes facing bile canaliculi.

The liver localization of TNAP suggests two different functions (Figure 1). First, in mouse liver TNAP is localized at hepatocyte and cholangiocyte membranes facing bile canaliculi and ducts, with some differences between mouse strains [12,13]. In cultured rat hepatocytes, TNAP activity is also seen at the bile canalicular-like membrane [18,19]. This suggests a role for TNAP in bile excretion (discussed in Section 2.2). Second, TNAP is also expressed in endothelial cells of hepatic arteries in the portal triad [13]. This suggests a role for TNAP in the transport of molecules from the blood to the liver (discussed in Section 2.4).

### 2.2. Function of TNAP in Bile Excretion

An important function of hepatocytes is to produce the bile, which consists of bile acids, the end-products of cholesterol metabolism, cholesterol itself, phosphatidylcholine and toxic metabolites [20]. It is highly conceivable that liver TNAP plays a role in bile excretion [3]. As mentioned above, TNAP is active at the hepatocyte and cholangiocyte membranes facing bile canaliculi and ducts [12]. Moreover, cholestasis is clinically associated with increased serum TNAP activity, in part due to the retrograde reflux of biliary TNAP [21], and in part to the de novo expression of liver TNAP [22]. TNAP expression in cholangiocytes is indeed increased after bile duct ligation [21], and in cultured rat hepatocytes, bile acids dose-dependently increase TNAP activity [23]. Furthermore, in cultured human HepG2 hepatocytes, taurine-conjugated cholic acid and chenodeoxycholic acid time- and dose-dependently increase TNAP activity [24].

**Figure 2:**
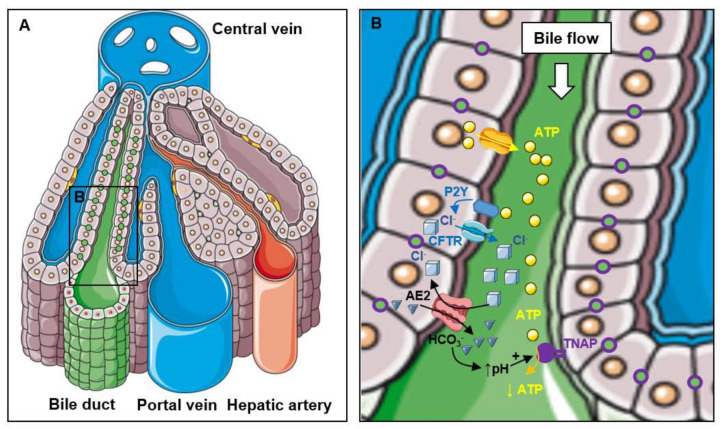
Function of TNAP in bile excretion (**A**) Rough presentation of liver structures indicating the region presented in (**B**). (**B**) Typical organization of hepatocytes delimitating bile canaliculi, showing from the top to the bottom the sequence of molecular events involved in pH regulation during bile excretion.

The most likely function of TNAP in bile excretion is to participate in the regulation of bile pH and of bile excretion during the postprandial period (reviewed in [3,25,26]). Cholangiocytes are exposed to millimolar concentrations of extracellular bile salts. Glycine and taurine conjugates of bile salts have a pK_a_ of approximately 4 and 1–2 respectively, and therefore, at these high concentrations, a significant amount of bile acids might be protonated and toxic [25]. The increase in bile pH from 7.3 during fasting to 7.5 after meal ingestion that accompanies bile secretion is therefore likely necessary to protect cholangiocytes [25]. How bile pH is regulated in association with bile excretion is relatively well understood (Figure 2). Exposed to bile flow [27], hepatocytes and cholangiocytes release adenosine triphosphate (ATP) in the bile to activate purinergic P2Y receptors and enhance Cl^−^ secretion through cystic fibrosis transmembrane regulator (CFTR). The rise of extracellular Cl^−^ then stimulates the Cl^−^/HCO_3_^−^ exchanger AE2 (anion exchanger 2) to allow carbonate secretion and increase the pH [3,25,28]. The hormone secretin also stimulates ductal choleresis by activating CFTR and subsequently AE2. Activated by the rise in pH, TNAP may then dephosphorylate ATP to reduce P2Y receptor activation, allowing the pH to reach back initial values (Figure 2). This hypothetical contribution of TNAP to pH regulation during choleresis was experimentally strengthened by Alvaro et al., who showed that AP administration in rats slowed down bicarbonate secretion and bile flow that were stimulated by secretin [21]. They moreover showed that TNAP inhibition with levamisole logically induced a significant increase in basal bile flow [21]. Interestingly, such a function of TNAP in the regulation of the gastrointestinal pH has already been described for intestinal alkaline phosphatase. Indeed, IAP is believed to regulate the intestinal pH and protect the mucosa against acidic injuries by dephosphorylating ATP [29]. In summary, TNAP may play a role in the regulation of bile excretion, and consequently of lipid digestion. Whether this role accounts for the liver steatosis in *Alpl*^+/−^ mice fed a high fat diet is unknown but deserves consideration [14].

### 2.3. Function of TNAP in the Bile and the Intestinal Lumen

The function of liver TNAP may not be restricted to bile excretion but might extend to intestinal digestion and absorption of lipids and/or bacterial compounds. A fraction of liver TNAP is indeed excreted in the intestinal lumen within the bile. TNAP is released with fragments of hepatocytes and/or cholangiocytes upon the action of bile acids [30]. In addition to liver TNAP, bone TNAP is also excreted in the bile after its release from bones into the blood. At the hepatocyte membrane, circulating TNAP binds to the asialoglycoprotein receptor, which recognizes galactose-terminated glycoproteins, and is transcytosed toward the bile [21,31]. Bone and liver TNAP seem to be excreted in the bile at different rates due to their different glycosylation pattern. While both isoforms are N-glycosylated, which seems important for their catalytic activity, bone, but not liver TNAP, is O-glycosylated [32,33]. Moreover, the liver isoform is more sialylated than the bone one, and is eliminated more slowly [31]. Therefore, some liver and bone TNAP activity is constantly released from inside the body to the intestinal lumen with bile excretion. The function of TNAP in the intestinal lumen is unknown. Since TNAP and IAP share a similar enzymatic activity (reviewed in [1]), it can be hypothesized that TNAP helps IAP accomplish its tasks.

**Figure 3:**
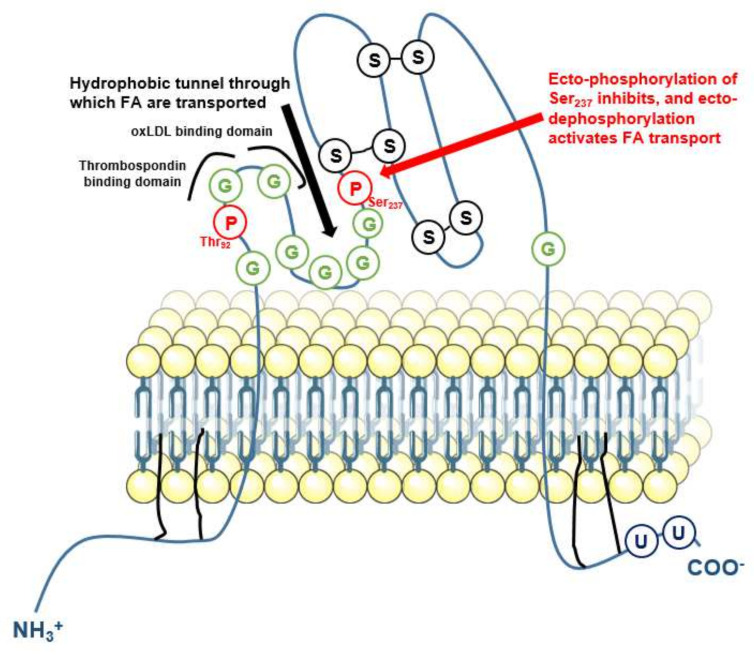
Schematic representation of CD36, showing its intracellular, transmembrane, and extracellular domains including posttranslational modification sites [47]. G: glycosylation sites (CD36 glycosylation is permanent and necessary for proper protein folding); P: phosphorylation site; S: cysteine involved in a disulfide bond; U: ubiquitination site.

IAP at least exerts two suspected important functions. First, IAP dephosphorylates bacterial lipopolysaccharide (LPS) in the intestinal track [34,35], to limit the postprandial endotoxemia that is inevitably associated with lipid absorption [36]. The 1′- and 4′-lipid A phosphates of LPS are considered critical for its pro-inflammatory effects as dephosphorylated lipid A congeners weakly stimulate toll-like receptor (TLR)-4 [37]. Accordingly, APs are thought to exert anti-inflammatory effects by dephosphorylating LPS and reducing TLR4 activation [38,39]. LPS dephosphorylation by IAP in the intestinal lumen is required to prevent intestinal, liver and systemic inflammation, metabolic syndrome, and early death [34,35]. IAP activity nevertheless decreases with recurrent episodes of infection, resulting in the progressive disability of the intestine to fight new infections [40]. The decline of IAP is associated with increased intestinal neuraminidase desialylation of IAP and increased IAP degradation, which in turns favors LPS activation of intestinal TLR4 [40]. Importantly, long-term oral supplementation with IAP in aged mice prolonged life expectancy with reduced systemic inflammation, liver damage, serum total cholesterol and triglycerides, reduced low density lipoprotein (LDL)-associated cholesterol (c-LDL), increased high density lipoprotein (HDL)-associated cholesterol (c-HDL), and reduced glycemia [35]. These beneficial effects likely relied on reduced LPS activation of TLR4 since they were attenuated in *Tlr4*-deficient mice [41]. In addition, in experimental liver disease induced by bile duct ligation or CCl_4_ exposure, oral IAP administration attenuated the compromised intestinal barrier permeability and liver fibrosis, in association with reduced LPS absorption [42]. Furthermore, intestine-specific overexpression of a chimeric alkaline phosphatase attenuated the metabolic syndrome induced by a western diet [43]. A second important function proposed for IAP is dephosphorylation of CD36 and modulation of fatty acid (FA) uptake [44,45]. CD36, which was first identified as a cellular receptor for thrombospondin and a protein involved in platelet aggregation, was subsequently discovered as a receptor for oxidized LDL and a facilitator of FA transport (reviewed in [46]). CD36 has two transmembrane domains and two consensus phosphorylation sites in its extracellular loop, at Thr92 and Ser237 (Figure 3, reviewed in [46]).

Thr92 is a consensus site for protein kinase C (PKC) and Ser237 for PKA [46]. In platelets, CD36 ectophosphorylation at Ser237 inhibits FA uptake, in a reversible manner after treatment with AP [48]. In intestinal cells, the transport activity of CD36 is also increased after treatment with IAP and decreased when IAP is inhibited [44]. Experimental data moreover exist to suggest that IAP and CD36 interact physically, which strengthens the hypothesis that IAP controls FA transport through CD36 phosphorylation status [45]. The fact that IAP-deficient mice have abnormal lipid transcytosis across enterocytes might account in the development of metabolic syndrome when these mice are fed a HFD [49]. Nevertheless, dephosphorylation of CD36 also increases thrombospondin binding [50], suggesting that AP dephosphorylation of CD36 might have other consequences than only modulating FA transport.

Whether TNAP that is eliminated in the duodenum within the bile exerts the same functions as IAP is unknown and remains purely speculative. On one hand, since mice deficient in *Akp3*, one of the two genes encoding IAP, have higher circulating TNAP levels than wildtype mice during aging [51], and since TNAP is eliminated in the intestine, it is tempting to speculate that this TNAP upregulation aims to compensate IAP deficiency. On the other hand, if TNAP and IAP have very similar enzymatic activities, TNAP is more effective in PP_i_ hydrolysis as compared to LPS, while the reverse is true for IAP [52]. This suggests that IAP and TNAP do not exert totally redundant activities. In addition, the fact that IAP activity was detected in the kidney [53], where AP activity is generally considered to rely exclusively on TNAP, also suggests that both enzymes are not redundant. Finally, the hypothesis that IAP and TNAP have relatively specific activities is strengthened by the fat that in inflammatory conditions, intestinal cells express both IAP and TNAP [54]. In the latter study, inflammation not only increased TNAP activity, but also modified its glycolysation and sensitivity to inhibitors, again suggesting that all AP do not have the same activity. Therefore, whether in the intestinal lumen TNAP helps IAP accomplish its functions or whether it exerts any specific effect remains speculative.

### 2.4. Functions of TNAP in Liver Metabolism

While the location of liver TNAP at the hepatocyte and cholangiocyte membranes facing bile canaliculi and ducts suggests a specific role in bile excretion and function, the presence of TNAP in endothelial cells of hepatic arteries in the portal triad suggests additional functions, likely associated with the dephosphorylation of circulating molecules [13]. Among these candidate molecules, it is highly conceivable that liver TNAP, together with the IAP that has been transferred from enterocytes to the blood after a fatty meal [55,56,57], contributes to dephosphorylate the absorbed LPS that has escaped from IAP in the intestinal lumen. Interestingly, circulating LPS is removed from the body into the bile, suggesting that liver TNAP is involved [58]. High endotoxemia is moreover associated with liver steatosis and fibrosis [59,60,61,62], as is TNAP deficiency [14]. The assumption that TNAP dephosphorylates and inactivates LPS in the blood is further strengthened by the report that the TNAP inhibitor levamisole increased the mortality of rats injected intraperitoneally with LPS [39]. In physiological conditions, endothelial TNAP is likely to account for the largest part of circulating LPS dephosphorylation, but in diseased livers, hepatocytes also likely participate. Indeed, while liver Kupffer cells appear to be the cells responsible for the removal of the largest part of LPS, hepatocytes seem also involved [58]. In rat liver sections, LPS was indeed efficiently dephosphorylated on hepatocyte and cholangiocyte membranes at locations where TNAP is normally active [63]. Moreover, it is noteworthy that TNAP activity is increased in hepatocytes during non-alcoholic steatohepatitis (NASH) development in association with an enhanced ability of fibrotic livers to dephosphorylate LPS [64]. We should also mention that TNAP in neutrophils might participate in postprandial LPS detoxification [65]. Neutrophils are recruited in the liver after a meal, in particular when it is enriched with fructose [66], and they accumulate in the liver of animals fed with high fat diets for several weeks [67]. High fat diets leading to the development of NASH are associated with metabolic endotoxemia [68], and chronic injections of low doses of LPS worsen inflammation and fibrosis during the development of experimental NASH [69]. Clinically, endotoxemia is also positively associated with NASH and liver fibrosis [60]. In this context, liver TNAP upregulation in NASH may be a mean to counteract LPS toxicity [64]. In summary, liver TNAP may contribute to reduce postprandial endotoxemia and postprandial inflammation, and its activation in NASH may be associated with the increased endotoxemia and inflammation featuring NASH. This function of TNAP may not be restricted to repeated copious high fat meals associated with absorption of large amounts of LPS [36]. Indeed, the group of Donath et al., reported that LPS-associated postprandial inflammation is necessary for optimal insulin secretion and regulation of postprandial hyperglycemia [70]. TNAP may thus help resolve this physiological and useful low-grade postprandial inflammation.

Like in the intestinal lumen, another possible TNAP substrate in the liver is CD36. In HepG2 hepatocytes cultured in presence of high glucose or FA levels, TNAP expression increased in parallel with that of CD36 [71,72], and TNAP inhibition with levamisole or siRNA reduced triglyceride (TG) accumulation [71,73]. That TNAP controls CD36 activity in hepatocytes however remains to be demonstrated. Since CD36 dephosphorylation facilitates FA uptake [44,45,46], if TNAP was responsible for dephosphorylating CD36, then its deficiency should lead to decreased, and not increased liver steatosis [14]. In fact, whether the function of TNAP in lipid accumulation in hepatocytes [71,72] relies on CD36 modulation remains obscure, because TNAP was reported to be located at the surface of lipid droplets [71], and thus not where CD36 is expected to be active. Finally, it is conceivable that TNAP indeed participates in CD36-associated FA uptake, but on other tissues than the liver, nevertheless impacting liver steatosis. Indeed, CD36 appears to be responsible for a large part of FA uptake in heart, skeletal muscle, adipose tissue, but not liver [74]. If TNAP deficiency in these tissues decreases in CD36-dependent FA uptake, and if liver uptake of FA does not significantly rely on CD36 [74], then TNAP deficiency may increase the amount of FA available for liver uptake through CD36-independent mechanisms. This speculative mechanism is supported by the fact that CD36-deficient mice have increased, and not decreased, liver TG [75,76]. In summary, the possibility that TNAP deficiency leads to steatosis in association with CD36 ectophosphorylation is uncertain but deserves consideration.

**Figure 4:**
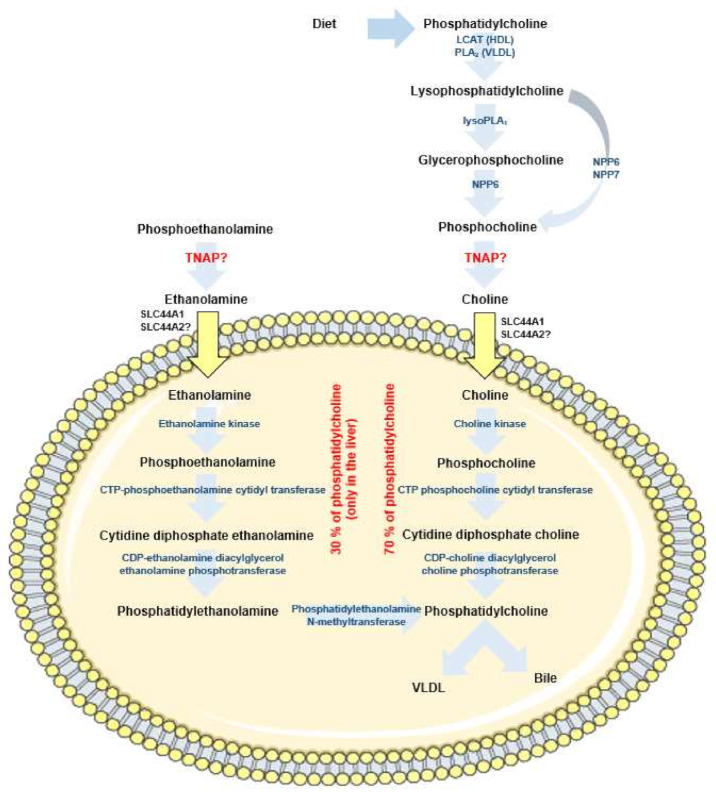
Sources of liver phosphatidylcholine showing the two main pathways allowing hepatocytes to produce phosphatidylcholine. On the right, phosphatidylcholine generation after the cellular uptake of choline arising from the diet or lipoproteins; on the left, generation of phosphatidylcholine from PEA. The phosphatase(s) generating choline and ethanolamine extracellularly are not known and may include TNAP. CDP: cytidine diphosphate; CTP: cytidine triphosphate; LCAT; lecithin cholesterol acyltransferase; NPP: nucleotide pyrophosphatase/phosphodiesterases PLA_2_; phospholipase A_2_.

Finally, the fact that *Alpl*^+/−^ mice develop an exacerbated steatosis as compared to *Alpl*^+/+^ mice when the steatosis is induced by a HFD, but not when the steatosis is induced by a choline deficient-diet [14] suggests a possible role in phosphocholine dephosphorylation. Phosphatidylcholine is necessary in the liver for the production of very low density lipoproteins (VLDL) and the excretion of bile, and choline deficient diets are commonly given to induce liver steatosis in rodents (reviewed in [77,78]). About 70% of liver phosphatidylcholine arise from the uptake of extracellular choline (Figure 4).

Phosphatidylcholine from the diet or from circulating lipoproteins is converted to phosphocholine by several enzymes that are still incompletely identified [77,79]. Phosphocholine seems to be generated by the action of the nucleotide pyrophosphatase/phosphodiesterases (NPP) NPP6 and NPP7 [80,81,82]. NPP6 is expressed in the liver and its deficiency leads to liver steatosis in mice [82]. If TNAP dephosphorylates phosphocholine, or contributes to dephosphorylate it, and allows choline uptake in the liver through SLC44A1 and SLC44A2 choline transporters [83,84], this would provide an explanation as to why TNAP-deficient mice develop liver steatosis on a HFD diet but not on a choline-deficient diet [14]. The second mechanism generating phosphocholine in the liver, and accounting for approximately 30% of phosphatidylcholine synthesis, is the conversion of phosphatidylethanolamine into phosphatidylcholine by the enzyme phosphatidylethanolamine *N*-methyltransferase (PEMT) [77]. *Pemt*^−/−^ mice fed a HFD develop non-alcoholic fatty liver disease (NAFLD) resulting from lack of sufficient phosphatidylcholine to export TG in VLDL [85,86], and cholestasis due to insufficient phosphatidylcholine for bile excretion [87]. How extracellular phosphoethanolamine (PEA) is generated and how it is dephosphorylated into ethanolamine is, to our knowledge, still obscure. However, the fact that individuals with TNAP deficiency accumulate PEA in blood and urine [88] strongly suggests that TNAP is involved in PEA dephosphorylation into ethanolamine, and perhaps in the subsequent ethanolamine uptake into liver cells through SLC44A1 and SLC44A2 transporters [84]. It should however be pointed out that TNAP may increase extracellular PEA levels by reducing the activity of the pyridoxal phosphate-dependent enzyme ethanolamine phosphate phospholyase, thereby increasing the intracellular levels of PEA and its extracellular export (reviewed in [89]). If nevertheless TNAP indeed participates in extracellular PEA dephosphorylation, then it would be involved in the two known pathways that generate phosphatidylcholine in the liver.

## 3. Pathophysiological TNAP’s Function in Adipocytes

It seems now accepted that adipose tissue expandability relies on both the hyperplasia of existing adipocytes and the formation of new adipocytes from mesenchymal progenitors (reviewed in [90]). These adipocytes comprise at least three cell populations. White adipocytes store FA within TGs, and release them in the circulation when the glycemia drops. Brown adipocytes use FA energy to generate heat in mitochondria thanks to their production of uncoupling protein 1 (UCP1). Finally, a new population of adipocytes, named beige or “brite” adipocytes, have been shown to share features with brown adipocytes but reside within white fat depots [90]. Interestingly, several recent articles showed that TNAP may participate in both the commitment of mesenchymal progenitors into adipocytes, and in their function, particularly in brown and/or beige adipocytes.

### 3.1. Function of TNAP in Adipocyte Differentiation

While historically, TNAP has been associated with the differentiation of mesenchymal progenitors into mineralizing osteoblasts and chondrocytes, relatively recent data suggest that it is already functional in mesenchymal stem cells (MSCs), and also involved in MSC commitment into other lineages, and in particular adipocytes (reviewed in [91]). Published data however report data difficult to reconcile, maybe relying on the different maturation stage of cells used in these studies. It was for instance shown that bone marrow stromal cells from *Alpl*^−/−^ or *Alpl*^+/−^ mice or from patients with hypophosphatasia generate more adipocytes than bone marrow stromal cells from corresponding controls [92,93,94], suggesting that TNAP prevents MSC commitment toward adipocytes. On the other hand, Esteve et al., reported that TNAP was particularly expressed in a subpopulation of beige adipocyte progenitors, where its chemical or molecular inhibition reduced the levels of markers associated with adipocyte differentiation (PPARγ2, ChREBP, UCP1, LPL), and decreased TG accumulation [95]. Similar findings were obtained in adipocyte cultures, with TNAP expression increasing with the differentiation of 3T3-L1 or primary adipocytes, and its inhibition with levamisole decreasing TG accumulation [73,96,97]. The substrates and mechanisms through which TNAP may impact adipogenesis are poorly known. *Alpl*^+/−^ MSCs appear to have higher levels of extracellular ATP than *Alpl*^+/+^ MSCs, suggesting that TNAP might inhibit adipocyte differentiation through ATP dephosphorylation and decreased activation of purinergic receptors [93]. On the other hand, in adipocytes, like in hepatocytes [71], TNAP was localized in association with lipid droplets, suggesting that TNAP may control adipocyte functions intracellularly [96,97]. In contrast with these studies, another article reported that in mature 3T3-F442A adipocytes, inhibition of TNAP activity with levamisole decreased lipolysis [98]. When cells were stimulated to trigger lipolysis in these experiments, TNAP was relocated from the surface of lipid droplets to cytoplasmic regions close to them [98]. Despite these puzzling data, the fact that TNAP-positive adipocytes are more abundant in obese than non-obese women [95] merits to further explore the functions of TNAP in adipocytes.

### 3.2. Function of TNAP in Thermogenesis

**Figure 5:**
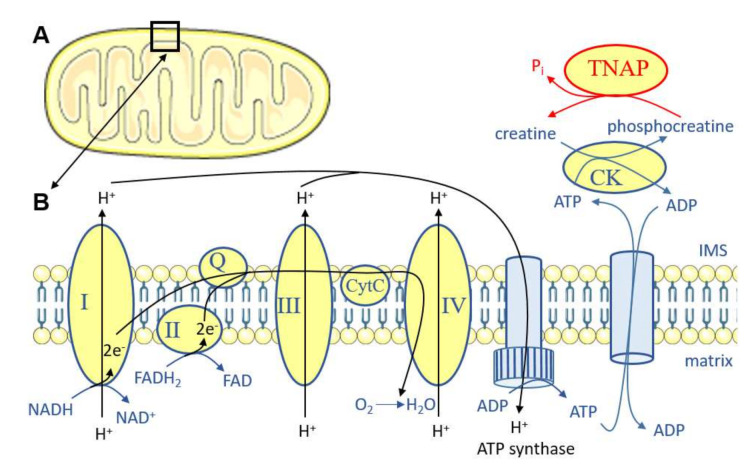
Schematic representation of the mitochondrial electron transport chain showing TNAP’s involvement in the futile creatine cycle. (**A**) indicates that the molecules and reactions presented in (**B**) are present at the inner mitochondrial membrane, in the intermembrane space, and possibly in the cytoplasm (it is still obscure where exactly TNAP may dephosphorylate phosphocreatine). I to IV represent complex I to complex IV. CK: creatine kinase; CytC: cytochrome C; FAD: flavine adenine dinucleotide; IMS: intermembrane space; NAD: nicotinamide adenine dinucleotide; Q: coenzyme Q.

Recently, TNAP in adipocytes was shown to participate in thermogenesis [99]. The most well-known mechanism contributing to heat generation is associated with the presence of UCP1 at the inner mitochondrial membrane of brown adipocytes. UCP1 generates heat by dissipating the energy proton gradient from the electron transport chain in mitochondrial respiration. Besides this function of UCP1 in brown adipocyte thermogenesis, additional UCP1-independent mechanisms generating heat have recently emerged, both in brown and beige adipocytes [100]. One of these mechanisms is the futile creatine cycle, characterized by the group of Spiegelman et al. [101]. This cycle is active in human fat and cultured brown and beige adipocytes and dissipates the energy of phosphocreatine by generating a P_i_ instead of an ATP [101] (Figure 5). The kinase responsible for phosphorylating creatine in adipocytes was shown to be creatine kinase (CK) B [102], and the phosphatase dephosphorylating phosphocreatine revealed to be TNAP [99]. Spiegelman et al., first observed that TNAP expression in adipocytes was increased after cold exposure, and that TNAP was unexpectedly localized near the inner mitochondrial membrane. TNAP inhibition decreased phosphocreatine dephosphorylation and respiration in vitro, and *Alpl* deletion in adipocytes in vivo induced obesity when mice were fed a HFD [99]. This suggests that TNAP in adipocytes, in addition to liver TNAP, plays an important role in the development of MetS.

Several important points remain to be explored after these pioneer studies on TNAP in adipocyte mitochondria. The first one is how TNAP localization to the cell membrane or to the mitochondria is controlled. To our knowledge, TNAP lacks classical mitochondrial addressing signals. Interestingly, an article published in this issue reports that TNAP is also likely associated with mitochondria in bone and muscle progenitor cells [94]. In these cells, TNAP inhibition increased mitochondrial respiration and ATP production [94], providing molecular evidence to understand why TNAP-deficient mice, sheep and humans have mitochondria with disorganized cristae and/or reduced muscle strength [15,94,103]. Whether these effects are associated or not with changes in the creatine phosphorylation status is unknown but deserves investigation. Additionally, deciphering whether TNAP can be dynamically relocated from a location to another or whether cell membrane TNAP and mitochondrial TNAP represent independent TNAP pools appears to be an important question to address. Finally, the second point to investigate is to what extent this function of TNAP in adipocyte mitochondria modulates the development of MetS, and in particular whether it impacts the development of steatohepatitis [14].

## 4. Conclusions

Circulating TNAP levels are associated with both the development of MetS and with the cardiovascular risk [4,5,6,7,8,9]. It is suspected that TNAP impacts cardiovascular mortality by increasing atherosclerotic plaque calcification [10]. However, recent data indicate that TNAP may exert important functions in several other tissues, such as the liver and adipose tissues (reviewed in [11]). Importantly, total or specific TNAP deficiency in mice fed a HFD exacerbates steatohepatitis [14] and obesity [99], indicating that TNAP may exert protective effects. Therefore, it will be crucial in future studies to take the whole spectrum of tissues and functions that are likely to be impacted by TNAP-targeting approaches into consideration to get a correct picture of TNAP’s functions in the development of MetS.

## Data Availability

Not applicable.
